# Characterization of the Metabolic Response of *Streptomyces clavuligerus* to Shear Stress in Stirred Tanks and Single-Use 2D Rocking Motion Bioreactors for Clavulanic Acid Production

**DOI:** 10.3390/antibiotics8040168

**Published:** 2019-09-27

**Authors:** David Gómez-Ríos, Stefan Junne, Peter Neubauer, Silvia Ochoa, Rigoberto Ríos-Estepa, Howard Ramírez-Malule

**Affiliations:** 1 Grupo de Investigación en Simulación, Diseño, Control y Optimización de Procesos (SIDCOP), Departamento de Ingeniería Química, Universidad de Antioquia UdeA, Calle 70 No. 52-21, Medellín 050010, Colombia; silvia.ochoa@udea.edu.co; 2 Bioprocess Engineering, Institute of Biotechnology, Technische Universität Berlin, D-13355 Berlin, Germany; stefan.junne@tu-berlin.de (S.J.); peter.neubauer@tu-berlin.de (P.N.); 3 Grupo de Bioprocesos, Departamento de Ingeniería Química, Universidad de Antioquia UdeA, Calle 70 No. 52-21, Medellín 050010, Colombia; rigoberto.rios@udea.edu.co; 4 Escuela de Ingeniería Química, Universidad del Valle A.A. Cali 25360, Colombia

**Keywords:** clavulanic acid, antibiotic, *Streptomyces*, bioreactors, shear stress, oxygen transfer, single-use, stirred tank, morphology, fed-batch

## Abstract

*Streptomyces clavuligerus* is a gram-positive filamentous bacterium notable for producing clavulanic acid (CA), an inhibitor of β-lactamase enzymes, which confers resistance to bacteria against several antibiotics. Here we present a comparative analysis of the morphological and metabolic response of *S. clavuligerus* linked to the CA production under low and high shear stress conditions in a 2D rocking-motion single-use bioreactor (CELL-tainer ^®^) and stirred tank bioreactor (STR), respectively. The CELL-tainer^®^ guarantees high turbulence and enhanced volumetric mass transfer at low shear stress, which (in contrast to bubble columns) allows the investigation of the impact of shear stress without oxygen limitation. The results indicate that high shear forces do not compromise the viability of *S. clavuligerus* cells; even higher specific growth rate, biomass, and specific CA production rate were observed in the STR. Under low shear forces in the CELL-tainer^®^ the mycelial diameter increased considerably (average diameter 2.27 in CELL-tainer^®^ vs. 1.44 µm in STR). This suggests that CA production may be affected by a lower surface-to-volume ratio which would lead to lower diffusion and transport of nutrients, oxygen, and product. The present study shows that there is a strong correlation between macromorphology and CA production, which should be an important aspect to consider in industrial production of CA.

## 1. Introduction

β-lactam antibiotics are the most widely used class of bioactive compounds for the treatment of infectious diseases. In the last century, thousands of penicillin derivatives and related β-lactam classes of cephalosporins, cephamycins, monobactams, and carbapenems have been developed to increase the spectrum of activity of those compounds [1]. Nevertheless, the inappropriate use of commercial antibiotics, e.g., through overdoses in medical treatments and the broad use of antibiotics as growth promoters in animal husbandry, has led to the massive development of resistance. This “antibiotic resistance crisis” is seen by the World Health Organization as one of the greatest threats to global health, food safety, and human development [2]. Resistance to β-lactam antibiotics is primarily due to the bacterial production of β-lactamase enzymes that hydrolyze the β-lactam ring, hence inactivating the drug [1].

Clavulanic acid (CA) is a β-lactam compound with a potent inhibitory activity on the β-lactamase enzymes. Pharmaceutical combinations of CA with amoxicillin or ticarcillin are widely used in clinical practice for the treatment of resistant infections. CA is produced by the gram-positive filamentous bacterium *Streptomyces clavuligerus* in submerged cultures under phosphate limitation. Shear conditions in CA production has been previously explored in airlifts [3] and stirred tank reactors [4]; however, the physiological response of *S. clavuligerus* to shear forces and its relationship with CA secretion is not completely understood. Many efforts have been carried out to increase the yield of CA since not only nutritional, but also environmental and degradation factors compromise the product yield in submerged cultivations of *S. clavuligerus* [5,6]. Several studies focused on the effect of nutrient concentrations and environmental cultivation conditions on CA biosynthesis, as Ser et al. [7] reviewed. Additionally, genetic engineering tools have been applied to *S. clavuligerus* aimed to improve the cellular metabolic capabilities for CA production in submerged cultures [8,9,10].

In microbial cultivations the effects of shear forces are particularly important since they affect the growth rate, broth rheology, and transport of nutrients, leading to specific morphological responses. Therefore, knowledge about the effect of shear stress on microorganisms of industrial interest is essential for the appropriate design and operation of the bioprocesses. The physiological and metabolic responses to shear stress are strain-dependent. In the case of *Streptomyces* sp., numerous studies have focused on the effects of nutritional factors on the production of antibiotics, but few studies have correlated hydrodynamic stress with antibiotic production, although these two conditions are strongly coupled in bioreactors [11]. The rocking-motion bioreactor is a form of technology that allows the investigation of the effect of low shear forces on macromorphology apart from oxygen starvation, which is not the case for STRs or airlift reactors at higher cell concentration. Additionally, the gas mass transfer is considerably higher than in the bubble columns, creating low shear stress but also low mass gas transfer. Additionally, antibiotic production in *Streptomyces* cultures also depends on an appropriate setup of the biophysical parameters e.g., pH, viscosity, agitation, and dissolved oxygen (DO), which affect morphological differentiation and metabolite secretion [12].

Some studies explored the impact of mechanical stress on *Streptomyces* morphology in suspension cultures, showing that high shear stress typically leads to the formation of small mycelial particles while clumping or pellet formation occurs at low shear stress [13,14]. Such pellets favor the accumulation of actinorhodin in *S. coelicolor*, nystatin in *S. noursei*, retamycin in *S. olindensis*, and nikkomycin in *S. tendae* as Manteca et al. [15] previously reviewed. In the case of *S. clavuligerus*, little is known about the relationship between shear stress, growth, morphology, and metabolite secretion. The dispersed mycelial morphology has been observed more often in *S. clavuligerus* cultivations performed in bioreactors and, in contrast to other phylogenetically related species, pellet formation seems to be related with a decrease in the CA production rate [16,17].

Single-use technology has emerged as an interesting alternative for biotechnological production of drugs due to its flexibility, scalability, availability of different stirring configurations, easy handling, reduced incidence of cross-contamination, and savings in operational costs and time [18,19]. Several single-use bioreactor types are currently commercially available [18,20]; the gas mass transfer of some of them is based on stirring, while it relies on shaking in others [21,22]. In particular, 2D rocking-motion bioreactors, which create a wave or cause eddy formation at the vessel wall for the suction of air into the liquid phase, exhibited comparably higher gas mass transfer rates while mechanical shear forces are considerably lower than in stirred tank bioreactors. This leads to an altered micromorphology and subsequently different growth and product synthesis rates as observed in cultivations performed in the two-dimensional rocking-motion bioreactor concept CELL-tainer® [19,23,24]. In this regard, the filamentous fungus *Aspergillus niger* (*A. niger*) cultivated in this low shear-force environment, showed a similar macromorphology as in shake flask cultures, while large pellets were formed. When talcum was added into the cultivation medium, a rather dispersed growth was achieved similar to the macromorphology obtained in stirred tank reactors [25]. While a fed-batch mode can be conducted, and the DO and pH-value can be controlled in a bioreactor more easily than in a shake flask, the effects of an altered shear stress on the cells macromorphology can be investigated in the 2D rocking-motion bioreactor independently from changes on gas mass transfer. If cultivations in a stirred tank reactor are compared with cultivations in 2D rocking-motion bioreactors, the relationship between shear forces, macromorphology and growth as well as product synthesis can be determined.

So far, to our knowledge, there are no reports in the literature addressing the effects of low shear forces in single-use systems on the physiology and morphology of *Streptomyces* species. The present study aims at performing a comparative analysis of the metabolic response of *Streptomyces clavuligerus* and the production of CA to the shear stress effects in a 2D rocking-motion single-use bioreactor (low-shear stress condition) and a stirred tank reactor (high-shear stress condition), since the hydrodynamic condition is an important parameter to consider in industrial production of CA. For acquiring an equitable comparison, fed-batch cultivations of *S. clavuligerus* were carried out under identical operating conditions in both bioreactors.

## 2. Results

### 2.1. Biomass and Morphology

**Figure 1:**
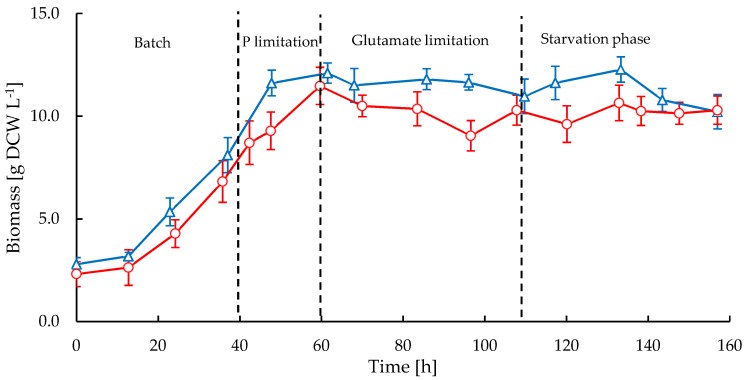
Time course of biomass in stirred tank bioreactor (STR, triangles) and CELL-tainer^®^ (CT, circles) in fed-batch cultivations of *Streptomyces clavuligerus.*

*S. clavuligerus* was cultivated at bench scale in stirred tank reactor (STR) and rocking-motion single-use bioreactor CELL-tainer^®^ (CT) reactors to investigate the impact of shear forces on macromorphology and CA production. Figure 1 shows the time course of biomass concentration during 6.5 days of cultivation in both reactors. Similar growth trends were observed in both cultivations, but higher growth rates were attained in the STR, showing also higher maximum biomass concentration (12.3 g·L^−1^ dry cell weight (DCW)) compared to CT cultivations where the maximum biomass was 11.5 g·L^−1^ DCW. The maximum specific growth rates for the STR and CT cultivations were 0.050 h^−1^ and 0.042 h^−1^, respectively. The exponential growth phase started at 14 h in both reactors and the stationary phase coincided with phosphate and glutamate starvation between 60 and 70 h. Only a small amount of phosphate (~0.47 mmol·s^−1^) was fed to the reactor during the fed-batch stage for maintenance without repressing CA biosynthesis. For both cultivations, a slight decline of biomass occurred from 133 h onwards and the final biomass concentration at 157 h was rather the same (10.2 g·L^−1^ DCW) in both bioreactors.

**Figure 2:**
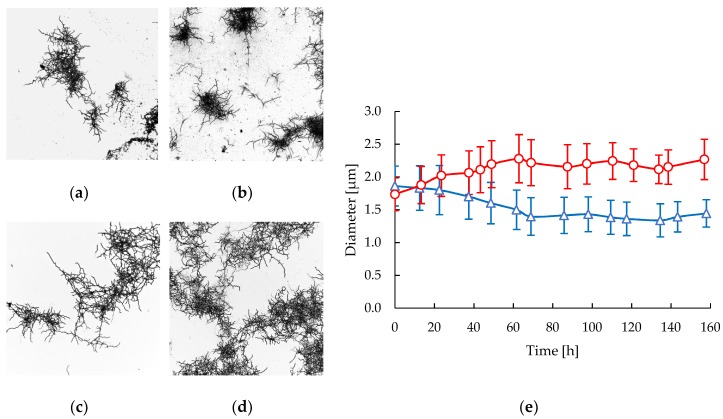
Morphological response of *S. clavuligerus* in STR (triangles) and CT (circles) bioreactors: (**a**) Mycelia in STR at 22 h; (**b**) Mycelia in STR at 86 h; (**c**) Mycelia in CT at 23 h. (**d**) Mycelia in CT at 87 h; (**e**) Time course of mycelial diameter in STR (blue) and CT (red).

In all the experiments performed in the STR and CT reactors a mycelial morphology was observed as the far dominant or only form. Highly fragmented and less branched mycelial structures were observed in the STR after 86 h of cultivation (Figure 2b) compared with the mycelia in the early stages of cultivation (Figure 2a). In the CT samples the preservation of macromorphology along the cultivation is notable (Figure 2c,d), showing less fragmentated, more branched, and slightly aggregated mycelia. In contrast to what was observed in the STR, a significant increment of filament diameter along the cultivation time was measured in CT samples (Figure 2e). A clear decrease in the filament diameter of 22.5% was measured in the STR, varying from 1.86 ± 0.30 μm to 1.44 ± 0.21 μm during the course of the cultivation. In contrast, the filaments diameter increased by 30.6% in CT cultivations in the same time, varying from 1.74 ± 0.25 μm to 2.27 ± 0.31 μm.

### 2.2. Shear Stress and Oxygen Transfer

**Figure 3:**
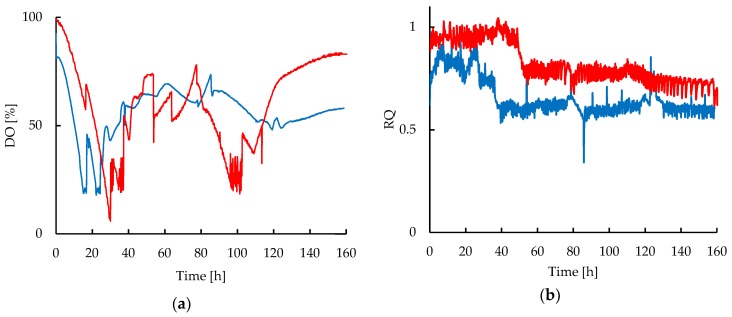
Dynamic profiles of oxygen in liquid and exhaust gas in fed-batch cultivations of *S. clavuligerus*: (**a**) Dissolved oxygen (DO) in STR (blue) and CT (red) cultivations; (**b**) Respiratory Quotient (RQ) in STR (blue) and CT (red) cultivations.

During the experiments, the DO was used as an indicator of oxygen availability in the medium and it was also selected as the control variable to assure the comparability of both cultivations even under different hydrodynamic conditions, as it can be observed in Figure 3a. Since the CT provided a high capacity to increase the DO in the media, only small changes of rocking velocity were applied when needed to maintain the DO values closer to those of the STR. A minimum set-point of 20% for the DO was defined for all cultivations, but it was preferable to maintain the DO between 50% and 70% by controlling the agitation speed between 300 and 500 rpm in the STR and 15 to 25 rpm in the CT, respectively. This was done in order to maintain moderate shear forces on the strain, avoiding extreme agitation values that could affect the cell or favor CA degradation.

**Figure 4:**
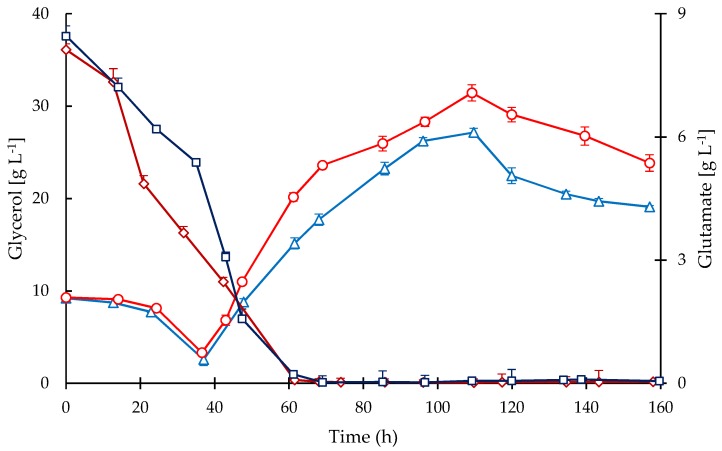
Time courses of substrates in the culture media for fed-batch cultivations of *S. clavuligerus.* Glycerol in STR (triangles) and CT (circles), glutamate in STR (squares) and CT (diamonds).

Table 1 shows the estimated volumetric oxygen transfer coefficient (k_L_a) values, the apparent viscosity (µ_app_) of the broth, and the maximum shear stress (τ_max_) at different mixing velocities (N). The mass transfer is limited by the increasing viscosity of the broth, therefore the requirement of increasing the agitation velocity to maintain DO and k_L_a values. Agitation in both reactor underwent a turbulent flow according to the calculated values of Reynolds number at the operating conditions. The maximum Reynolds numbers at 500 rpm in STR and 22 rpm in CT were 13,582 and 15,896, respectively. The k_L_a values were considerably higher in the rocking-motion reactor than those attained from the STR, during all experiments. Therefore, only small changes in agitation speed in the CT showed a significant increase in the k_L_a and DO values. Interestingly, it was also observed that despite the differences in the volumetric oxygen transfer attained in both reactors, the growth rate of *S. clavuligerus* was not significantly affected by an enhanced oxygen transport. The Respiratory Quotient (RQ) (see Figure 3b) was close to 1 during the early stages of cultivation. After 40 h for STR and 50 h for the CT cultivations the RQ decreased, presumably linked to glutamate and phosphate depletion during glycerol feeding. Despite the observation that the reduction of RQ occurred in both reactors, in the CT the RQ remained 15% higher after glutamate exhaustion in comparison with the STR. The lower RQ in the STR cultivations during the fed-batch phase coincided also with comparatively higher glycerol uptake, as shown in Figure 4.

*S. clavuligerus* fermentation broth is characterized by high viscosity, exhibiting rheological properties of a pseudoplastic fluid according to Equation (1). The mean behavior index (n) of the fermentation broth is 0.318 and the consistency index (K) depends on biomass concentration and shear conditions, ranging between 0.1 and 1.5 Pa s^n^. As presented in Table 1, the initial apparent viscosity of the broth, defined by Equation (2), was rather close to the value of water at the operating temperature (0.001 Pa s); then increased with cellular growth. The shear stress values (τ_max_) in the STR reactor were in the range from 1.4 to 7.6 Pa. In the case of CT the shear stress ranged between 0.06 and 0.63 Pa.

τ = Kγ^n^ (1)

µ_app_ = Kγ^(n − 1)^ (2)

### 2.3. Substrates and Products

All cultivations were operated in fed-batch mode suppling glycerol as the main carbon source and following a constant feeding pattern of 35 mL·h^−1^. Figure 4 shows the time courses of glycerol and glutamate concentrations in the culture media in both bioreactors. The glycerol consumption was mainly correlated with biomass accumulation, showing a rapid depletion after 14 h, i.e., after starting the exponential phase in the batch stage. Since glycerol is fed in excess, during the fed-batch stage a constant accumulation of glycerol was observed from 37 h onwards up to 110 h when the feeding was stopped. After 110 h a sustained decline in glycerol occurred indicating that even during the stationary phase the metabolic activity was rather high, which agrees with the observed continuous CA secretion until the end of the cultivations.

**Figure 5:**
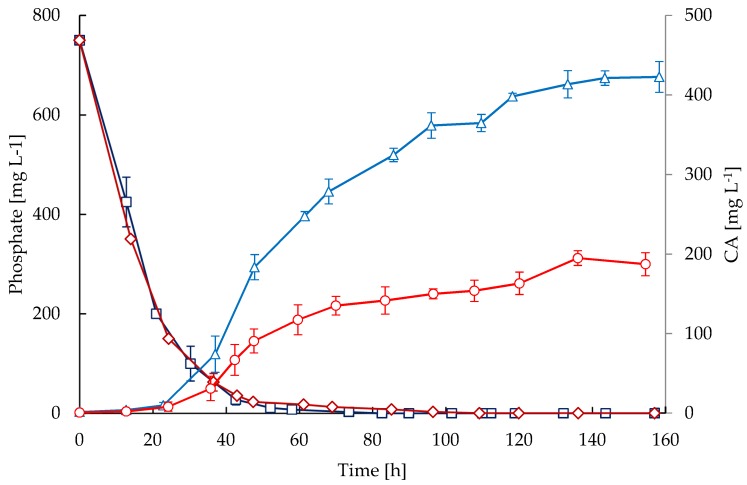
Time courses of clavulanic acid (CA) and phosphate in fed-batch cultivations of *S. clavuligerus.* CA in STR (triangles) and CT (circles), phosphate in STR (squares) and CT (diamonds).

A high concentration of glutamate was provided initially in the media but not in the feedings. Therefore, a rapid decline of glutamate concentration (Figure 4) was observed during the exponential phase in both bioreactors and it remained up to 70 h of cultivation; during this phase the glutamate was depleted. Similar to glycerol, glutamate uptake was higher during the first 45 h of cultivation in STR, which is congruent with a higher growth rate and biomass concentration. Note that growth rate (Figure 1) declined in both cultivations when glutamate became exhausted (Figure 4); in contrast, the accumulation of CA was not affected by glutamate starvation as observed in Figure 5. Although the nutritional conditions in both reactors were similar regarding glycerol, glutamate, ammonium, and phosphate, the accumulation of CA was considerably lower in CT cultivations. The secretion of CA was triggered after 24 h of cultivation, coinciding with a drop in phosphate concentration of at least 80%. Then, the CA concentration increased throughout the cultivation time and the maximum accumulation was obtained at the end of the process (157 h). The maximum mean concentrations of CA attained were 187.2 mg·L^−1^ for the CT and 422.7 mg·L^−1^ for the STR cultivations. The maximum CA specific production rates were 0.697 and 0.358 mg·(g DCW)^−1^·h^−1^ in the STR and CT, respectively.

**Figure 6:**
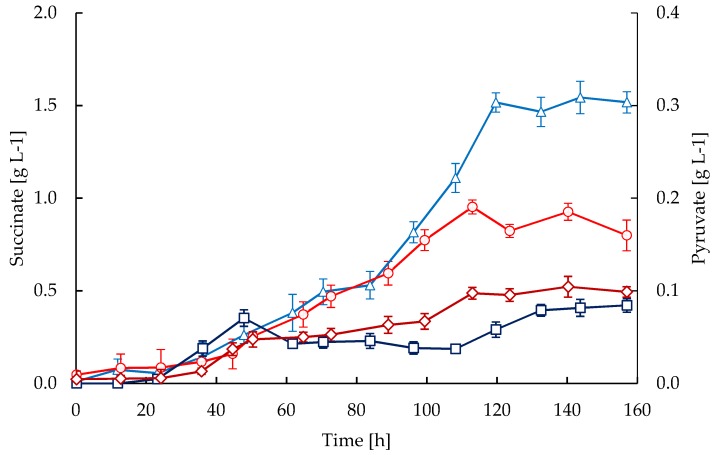
Time courses of succinate and pyruvate accumulations in fed-batch cultivations of *S. clavuligerus.* Succinate in STR (triangles) and CT (circles), pyruvate in STR (squares) and CT (diamonds).

Pyruvate and succinate were observed to accumulate differently in the STR and CT cultivations (Figure 6). The CT cultivations showed higher pyruvate levels compared to the STR. In both cultures the concomitant accumulation of succinate and CA were observed (Figure 5 and Figure 6), although succinate levels were considerably higher in the STR compared to the CT cultivations.

## 3. Discussion

Previous studies have extensively explored different cultivation conditions for promoting *S. clavuligerus* optimal growth and enhanced CA production. However, given the relationship between morphology and secondary metabolite secretion in the *Streptomyces* genus, a better understanding of the factors influencing the morphology in submerged cultures is required for the identification of optimal bioprocessing conditions. This has a special importance also for a later scale up of the process to an industrial environment, as the fluid dynamic conditions change with scale.

It is known that glycerol is the most suitable carbon source for *S. clavuligerus*, supplying glyceraldehyde-3-phosphate as an early C-3 precursor and substrate for energy metabolism and growth [26]. Thus, a constant feeding of carbon source was implemented to provide glycerol in excess for growth and maintenance, so as to promote CA biosynthesis. Additionally, a high initial concentration of glutamate was used in the production medium as a secondary carbon and nitrogen source, although it played an important role as an early C-5 precursor favoring the metabolic fluxes through amino acids metabolism and the urea cycle during the exponential growth phase. Note that growth rate (Figure 1) declined in both cultivations when glutamate became exhausted (Figure 4) despite the high availability of glycerol. This is the time that we interpret as the beginning of the stationary phase. This observation clearly indicates the importance of glutamate as a precursor of biomass when using a defined media.

*S. clavuligerus* is a strictly aerobic organism; thus, the oxygen transfer to the liquid medium in the reactor is a critical parameter to ensure proper growth and productivity [4]. In streptomycetes, the production of antibiotics is strongly affected by the balance of nutrients and oxygen availability in the medium, which can affect the growth rate, the induction of secondary metabolism, and maximum concentrations of secondary metabolites [11]. In this regard, determination of the k_L_a in bioreactors is an important parameter when assessing the oxygen mass transfer characteristics in connection with mixing and reactor geometry. In STRs, the increase of k_L_a implies vigorous stirring to favor mass transfer from gas to liquid phase by increasing the turbulence of the system, thus increasing shear stress on the medium and cells [27,28]. In contrast, wave and rocking-motion reactors are characterized by up to 100-fold lower shear forces compared to STRs [21,22,29] and especially 2D rocking-motion bioreactors provide higher k_L_a values compared to simple wave systems that are comparable to those obtained in STRs at high stirring speed [23,24,25].

As shown in Table 1, lower shear stress was experienced by the liquid phase and the cells of *S. clavuligerus* cultivated in the CT in comparison with the STR. The predominant filamentous morphology in liquid cultures of *Streptomyces* leads to an increase in broth viscosity and appearance of non-Newtonian rheology concomitant with cell growth [11]. The average shear stresses estimated for the fermentation broths were 0.44 and 4.92 Pa for the CT and STR, respectively. The oxygen transfer from the gas to the liquid phase is clearly favored by the 2D wave pattern generated during rocking motion. Nevertheless, the effect of oxygen mass transfer on the secondary metabolism of *S. clavuligerus* is not completely clear. The activities of deacetoxycephalosporin C synthase and isopenicillin-N synthetase increase with oxygen saturation and therefore the production of cephamycin C and penicillin N [30], while oxygen limitation (~10% DO) leads to inhibition of CA production [4]. Considering the requirement of molecular oxygen during CA biosynthesis, an operational window regarding DO values between 20% and 80% can be assumed as suitable for CA production, since the repression of CA at low DO values and the activation of the penicillin, cephalosporin and cephamycin biosynthesis occur when operating at oxygen saturation [4,30]. An increase of reaction fluxes through penicillin and cephalosporins pathways would potentially reduce the carbon flux toward CA biosynthesis due to an increasing demand of amino acid precursors.

The shear stress values (τ_max_) in the STR reactor presented in Table 1 were consistent with results from previous studies on cultivation of filamentous organisms in stirred tank bioreactors [3,31]. Shear stress higher than 6 Pa, as observed in STR reactors, triggers stress responses in sensitive strains like CHO cells. Nevertheless, the limits for cell damage and lysis are in the order of 100 Pa [32]. Previous studies have shown that *S. clavuligerus* can be cultivated in STR reactors at mixing velocities up to 800 rpm without compromising cell viability [33,34]. In contrast with STR, it has been widely reported that shear forces in rocking and wave reactors are considerably lower than those arising in reactors with axial or orbital agitation [29,35], which is also in agreement with our results considering the calculated maximum shear stress ranging between 0.06 and 0.63 Pa. For the sake of comparison, the typical shear stress for water as a fluid model in wave reactors is in the range of 0.01 to 0.1 Pa at agitation rates from 15 to 30 rpm [29,35]. The shear stress in the fermentation broth is not just a function of the shear rate of mixing; an increasing viscosity drastically changes the shear stress conditions to which the cells are exposed [36,37]. Therefore, the shear forces in *S. clavuligerus* fermentation broths were expected to exceed the values reported for Newtonian fluids at similar agitation conditions in STR and single-use reactors [22,29,35].

It is well known for *S. clavuligerus* that cultivation under intensive agitation and optimal nutritional conditions lead to the prevalence of mycelial morphology [38]. Exponential growth of *Streptomyces* in mycelial form takes place by a combination of tip growth and branching of multicellular hyphal sections connected in compartments [39,40]. Under mechanical stress the adhesive forces between hyphae may be weakened making the filaments prone to fragmentation [14]. Thus, intense agitation causes fragmentation, resulting in the formation of short hyphae capable of growing and reproducing under nutrient availability; otherwise, those fragments would eventually die [11,13]. 

The higher biomass production and apparent viscosity observed in STR (Figure 1) compared with the CT in our opinion are a consequence of the extensive hyphal fragmentation caused by the mechanical shear forces, which led to a great number of viable hyphal fragments. Additionally, the enhanced oxygen transfer entailed a positive effect on growth, possibly compensating for the potential shear damage caused by agitation nearby the impeller or reactor wall. In contrast, under mild agitation the individual hyphae tend to become longer and branched and slightly aggregated, resulting in a decrease of mass and heat transfer [11,13].

The macromorphology and product biosynthesis in submerged cultivations of filamentous organisms represent a multifactorial process affected by the bioprocessing conditions [25]. Several *Streptomyces* species have a marked tendency to aggregate due to the bioadhesive properties of hyphae; such is the case for *S. avermitilis* [41], *S. lividans* [14], *S. toxytricini* [42], and *S. coelicolor* [15], among others. Furthermore, the morphological changes associated with the increase in shear stress are related to different physiological activities [13]. Specifically, in streptomycetes the production of many secondary metabolites is affected by agitation frequency and power dissipation, suggesting a possible relationship between agitation, micromorphology, and product secretion [11]. An increase of the agitation rate in the range from 200 to 600 rpm was observed to favor the production of neomycin in STR cultivations of *S. fradiae,* but a negative effect was reported for agitation values above 600 rpm [43]. Similarly, natamycin production by *S. natalensis* increased up to 7-fold when the agitation was increased from 50 to 250 rpm in an STR [44,45]. In shake-flask cultures of *S. pristinaespiralis*, an increase in power dissipation between 2 and 5 kW m^−3^ increased pristinamycin accumulation [46]. For all cases, a threshold in the yield was observed when the agitation rate was increased, indicating that cell damage and loss of viability might occur when exceeding the tolerable agitation limits [11].

We observed thinner and dispersed mycelia at high shear stress in the STR and this was coincident with a high CA productivity. During the lag and early exponential phases no statistical differences were observed in the morphology of *S. clavuligerus* cultivated in either the STR or CT reactors despite the significant difference in shear forces. The increase of agitation speed from 300 to 500 rpm in the STR led to an increase of shear stress, thus causing a sustained decrease of mycelial diameter during the exponential growth (Figure 2e) and a higher growth rate, in comparison with the CT cultivations. Conversely, the mycelial morphology in the CT cultivations suggests that a reproduction mechanism of elongation, branching and thickening of existent mycelia is favored under low shear forces, also promoting slight aggregation due to adhesive properties of the hyphae [14]. In contrast, continuous fragmentation in the STR avoids reaching significant elongation, thickening and branching of mycelia. Interestingly, morphological differentiation induced by the hydrodynamic regime was observed to stop when glutamate was exhausted, coinciding with the start of the stationary phase. Therefore, the filament diameter acquired up to this point (60 h), remained rather constant during the rest of the cultivations as shown in Figure 2e.

It is known that antibiotic production in streptomycetes is initiated by stress conditions e.g., essential nutrient limitations [13,39]. Although the nutritional conditions in both reactors were similar regarding glycerol, glutamate, ammonium, and phosphate, the accumulation of CA was considerably lower in CT cultivations. It has been widely reported that phosphate is a strong repressor of CA biosynthesis [3,4,26,47]; indeed, the secretion of CA was triggered after 24 h of cultivation, coinciding with a drop in phosphate concentration of at least 80%. According to our observations, CA production was enhanced by the shear conditions present in the STR. Although nutritional conditions were the same in the cultivations performed in both reactors and phosphate limitation triggered the CA secretion at approximately the same time, the CA production rate in STR was 2.5-fold the observed in the CT cultivations. Presumably two factors are linked to the observed low CA biosynthesis: (1) under the environmental conditions of the CT reactor, cellular activity might be catabolizing nutrients for growth regulation through mycelial thickening and branching, leaving, as a consequence, a smaller net amount for cell production, as indicated by the lower specific growth rate observed; and (2) the difference in the RQ values observed in CT and STR reactors suggests a limited oxygen transfer to the intracellular environment in the CT cultivations, probably due to adhesion of the more branched filaments and lower surface to volume ratio given the observed mycelial thickening, leading to higher RQ values in comparison to those observed in the STR. Similar observations regarding the secretion of secondary metabolites and RQ values linked to filament aggregation have been reported for the filamentous organism *A. niger* cultivated at low shear forces [25]. Additionally, a lower uptake of glycerol and glutamate were also observed in the CT cultivations, suggesting that CA production may be affected by the lower surface-to-volume ratio of mycelia which would lead to lower diffusion and transport of nutrients, oxygen, and product secretion.

Additionally, intracellular availability of oxygen plays a key role in CA biosynthesis, considering the oxidation steps catalyzed by the enzyme clavaminate synthase [48]. According to Li et al. [49], the RQ values close to 1 observed in both reactors during the first hours of cultivation (see Figure 3b), indicate a balanced metabolism consistent with the aerobic oxidation of glycerol and glutamate as carbon sources present in the media. During the fed-batch operation the appearance of phosphate limitation led to a rapid decline in the RQ showing the increase of oxygen demand in this stage. The increase in glycerol concentration during the feeding added to the phosphate and glutamate depletions led to a metabolic imbalance between the glycolysis and the tricarboxylic acid (TCA) cycle, accompanied by an increase in the glycerol uptake since it is the only carbon source available in the media. The latter favors the substrate level phosphorylation as an alternative for ATP production under phosphate limitation [50].

It has been previously reported that activation of secondary metabolism under phosphate limitation in streptomycetes is also accompanied by a decline in the RQ [51]. Therefore, the accumulation of intermediates derived from the metabolic imbalance between glycolysis and the TCA cycle would promote the biosynthesis of antibiotics [51,52]. In our case, the decline in RQ was accompanied by a slight decrease in the specific growth rate (Figure 1), and the accumulation of pyruvate and succinate, respectively (Figure 6). Previously, Ramirez-Malule et al. [16] also reported the accumulation of succinate during CA production in 0.3 L of continuous cultivations under different feeding conditions; however, pyruvate was not observed to be accumulated under such conditions. According to Viollier et al. [52] the secretion of glycolytic and TCA intermediates in *Streptomyces* evidences the metabolic imbalance in the central metabolism. Moreover, the trends in the accumulation of pyruvate and succinate were opposite in the CT and STR reactors. The higher accumulation of pyruvate in CT cultivations (Figure 6) might be related to a catabolic repression as a consequence of the reduced oxygen uptake, which would lead to a decrease in the reaction fluxes along the TCA cycle and hence, in the respiratory chain. The activation of CA biosynthesis in *S. clavuligerus* is expected to increase the demand for oxygen as occurred in STR cultivations, since the reactions catalyzed by the clavaminate synthase enzyme require molecular oxygen for the oxidation of 2-oxoglutarate to succinate. This explains why CA biosynthesis led to a significant accumulation of succinate in our STR cultivations with higher CA productivity.

Interestingly, the decrease in the RQ was considerably less pronounced in the CT cultivations corresponding with the lower oxygen and glycerol uptake during the fed-batch stage, which also coincided with a lower CA biosynthesis rate compared with the STR cultivations. Indeed, adhesion of mycelia in *Streptomyces* limits the mass transfer of nutrients and reduces the oxygen uptake as a consequence of a higher resistance to diffusion, which not only affects the growth, but also the antibiotic production [11,42]. In contrast, the looser and thinner mycelial structures of *S. clavuligerus* cultivated at high shear conditions impose less resistance to oxygen diffusion from the medium to the intracellular compartment, allowing the cultures to effectively respond to the higher demand for oxygen, in accordance with the RQ values, which ranged from 0.5 to 0.7 during CA production in STR. These results suggest the RQ as an additional parameter to consider in *S. clavuligerus* cultivations for CA production in connection with the macromorphology and the hydrodynamic conditions. In this regard, an online RQ-control approach could be applied to *S. clavuligerus* as a strategy to promote changes in carbon fluxes and therefore, the synthesis of the desired products [49,53].

## 4. Materials and Methods 

### 4.1. Strain and Cultivation Procedures

*S. clavuligerus* DSM 41826, stored at −80 °C in a glycerol solution (16.7% *v/v*), was inoculated for activation in seed medium as described by Roubos et al. [54]. Two cultivation cycles (seed and preculture) were carried out prior to reactor inoculation. Cryotube cell suspensions (1.2 mL) were inoculated into 50 mL of seed medium in a 250-mL Ultra-Yield™ shake flask (Thomson Instrument Company, Oceanside, CA, USA). Cells were grown in a rotary shaker incubator for 26 h at 200 rpm and 28 °C. For the preculture, 2500 mL Ultra Yield shake flasks were filled with 450 mL of chemically defined medium, inoculated with 50 mL of cultivated seed broth. Cells were grown for 20 h using identical conditions [16].

Fed-batch cultivations were carried out in duplicate in a 15 L stirred tank bioreactor (STR), (Techfors S, Infors AG, Bottmingen, Switzerland) and in a 20 L single-use 2D rocking-motion bioreactor CELL-tainer^®^ (CT), (Celltainer Biotech BV, Winterswijk, The Netherlands); both operated at 5-L initial filling volume. Bioreactors were inoculated at 10% *v/v* from preculture. A chemically defined medium, formulated as follows, was used (per L): glycerol (9.3 g), K_2_HPO_4_ (0.8 g), (NH_4_)_2_SO_4_ (1.26 g), monosodium glutamate (9.8 g), FeSO_4_·7H_2_O (0.18 g), MgSO_4_·7H_2_O (0.72 g), and trace elements solution (1.44 mL). The trace elements solution contained (per L): H_2_SO_4_ (20.4 g), monosodium citrate·1H_2_O (50 g), ZnSO_4_·7H_2_O (16.75 g), CuSO_4_·5H_2_O (2.5 g), MnCl_2_·4H_2_O (1.5 g), H_3_BO_3_ (2 g), and Na_2_MoO_4_·2H_2_O (2 g) (all from Carl Roth GmbH, Karlsruhe, Germany). Antifoam 204 (Sigma Inc., St. Louis, MO, USA) was used at concentration of 1:1000 *v/v*, pH was controlled at 6.8 by using 4 M solutions of either NaOH or HCl. Aeration was provided at 0.6 vvm and temperature was controlled at 28 °C. Reactors were equipped with pH (Polylite Plus) and dissolved oxygen (DO, VisiFerm) probes (Hamilton Inc., Bonaduz, Switzerland). Outgas analysis was performed by O_2_ and CO_2_ gas sensors coupled to an exhaust gas analyzer (BlueInOne Ferm, BlueSens GmbH, Herten, Germany).

Batch operation was carried out during the first 37 h of cultivation, followed by fed-batch operation during the next 77 h. The feed medium had the following composition (per L): glycerol (120.0 g), K_2_HPO_4_ (2.0 g), (NH_4_)_2_SO_4_ (8.0 g); the feeding rate was set at 35 mL·h^−1^. The batch and fed-batch media were designed for achieving phosphate limitation around 40 h considering the phosphate/carbon ratio of biomass composition [16]. Once the fed-batch stage ended, cultivation continued without any feeding for 43 h (total cultivation time of 157 h). Agitation was controlled manually in the range of 300–500 rpm in the STR and 12–25 rpm in the CT; DO was maintained between 50% and 70%, avoiding the sudden oscillations of agitation originated by the DO automatic control. Culture samples (2 mL) were taken at 12-h intervals and centrifuged at 15,000 rpm and 4 °C for 10 min; wet biomass was washed with 0.9% NaCl and centrifuged. Test tubes were dried overnight at 75 °C for dry cell weight (DCW) determination.

The rheological parameters of the broth were determined according to Campesi et al. [37] and Cerri & Badino [3]. The k_L_a was calculated using the stationary method from exhaust gas data analysis [24,25]. The estimation of shear stress in both reactors was performed according to the methodology established for STR [36,55,56,57] and correlation of data for single-use bioreactors [29,35,58].

### 4.2. Mycelium Measurement and Product Quantification

The time courses of changes in mycelial diameter was followed by random measurement of individual mycelia for each cultivation sample and replicates. *S. clavuligerus* mycelia directly taken from cultivation samples were stained with crystal violet, observed, and photographed using a Nikon Eclipse Ti2 inverted microscope (Nikon Instruments Inc., Amsterdam, The Netherlands) at 400× magnification in order to capture a wide field of the sample. Post-processing of images and measurement of mycelial diameter were performed in ImageJ by applying digital zoom up to 250% and using the built-in measurement tool (NIH, Bethesda, MD, USA) [59]. Four different pictures were observed for each sample and at least 100 measurements of individual mycelia were performed per picture. Then, the mean values and standard deviations (SD) of mycelial diameter for each time point were calculated.

Quantifications of CA concentration in supernatant samples were carried out by HPLC equipped with a diode array detector (DAD, 1200 Series, Agilent Technologies GmbH, Waldbronn, Germany), using a Zorbax Eclipse XDB-C-18 chromatographic column (Agilent Technologies) and a C-18 guard column (Phenomenex^®^ GmbH, Aschaffenburg, Germany) operated with a flow rate of 1 mL/min at 30 °C. The mobile phase consisted of H_2_PO_4_ (50 mM, pH 3.2) and methanol (HPLC grade). HPLC analyses were performed by using the gradient method described by Ramirez-Malule [60]. Imidazole was used for derivatization of CA. The clavulanate–imidazole chromophore was detected at 311 nm. 

Glycerol, pyruvate, and succinate were quantified with an Agilent 1200 Series HPLC system equipped with a refractive index detector (RID) and operated at 15 °C using a HyperREZ™ XP carbohydrate H+ column (Thermo Scientific, Waltham, MA, USA) at a constant flow rate of 0.5 mL/min using 5 mm sulfuric acid solution as mobile phase [61]. Quantification of glutamate was performed with an Agilent 1260 Series Infinity HPLC system (Agilent Technologies), equipped with a fluorescence detector (FLD) with an excitation wavelength of 340 nm and emission wavelength of 450 nm. o-Phthaldialdehyde was used for precolumn derivatization of samples. A C18 Gemini® column with a SecurityGuard™ precolumn (Phenomenex) were used, operated at a flow rate of 1 mL/min and 40 °C. The mobile phase consisted of NaH_2_PO_4_ (40 mm, pH 7.8) as polar eluent and a solution of methanol (45 vol.%),.), acetonitrile (45 vol.%) and water (10 vol.%) as nonpolar eluent [62]. The semi-quantitative determination of phosphate and ammonium ions was performed by using phosphate and ammonia tests (MQuant™; Merck KgaA, Darmstadt, Germany). Statistical analysis of results was performed in terms of standard deviations and averages.

## 5. Conclusions

The results of this study indicate that low shear forces did not lead to significant hyphal fragmentation or lysis in *S. clavuligerus*; on the contrary, it promoted mycelial thickening and branching mechanisms of growth, hence favoring the preservation of macromorphology during the cultivation. The motion pattern of the rocking-motion single use bioreactor CELL-tainer^®^ is able to provide high values of volumetric mass transfer coefficient, facilitating oxygen dissolution even at low rocking speeds while exerting low shear stress on the cells and the liquid. Under low shear forces in the CELL-tainer^®^ the mycelial diameter increased considerably (average diameter 2.27 in CELL-tainer^®^ vs. 1.44 µm in STR). 

In addition to phosphate limitation, oxygen transport from the liquid phase to the cells also seems to be a critical factor for CA biosynthesis. Thickening of the mycelium reduces the surface-to-volume ratio, therefore limiting the oxygen transport and leading to a decrease in the oxygen uptake, which consequently results in a low production rate of CA. Thus, the high shear forces attained in the stirred tank bioreactor prevented mycelial adhesion and promoted high uptakes of glycerol and oxygen required for CA production. Besides, shear stress might have caused stress responses on the omics level also contributing to the secondary metabolite secretion, which remains to be investigated. The notable production of CA observed at RQ values between 0.5 and 0.7 suggests that RQ is a useful parameter for obtaining information about the oxidative metabolism of the bacteria and activation of secondary metabolism.

Here we presented a methodology for the analysis of the effect of shear forces on the bioprocess performance of *S. clavuligerus* without introducing other disturbances to the process, allowing the study of the physiological and metabolic response of the strain to the environmental shear conditions. The results can be valuable for other studies focused on strain and process optimization. Additionally, further application of an online RQ-control approach would help to redistribute carbon fluxes on primary metabolism and eventually contribute to increase the product synthesis.

## Figures and Tables

**Table 1:** Biomass as dry cell weight (DCW), culture volume (V), agitation velocity (N), volumetric mass transfer coefficient (k_L_a), maximum shear stress (τ_max_), and apparent viscosity (µ_app_) of fermentation broths in STR and CT cultivations.

Bioreactor	DCW (g·L^−1^)	V (L)	N (rpm)	k_L_a (h^−1^)	τ_max_ (Pa)	µ_app_ (Pa s)
CT	0.5	5.0	12	73.25 ± 7.40	0.066 ± 0.010	0.0011 ± 0.0005
	2.1	5.0	15	43.90 ± 0.92	0.102 ± 0.068	0.0012 ± 0.0001
	3.4	5.0	17	61.71 ± 8.13	0.222 ± 0.016	0.0021± 0.0013
	4.7	5.1	20	80.52 ± 8.86	0.147 ± 0.041	0.0041 ± 0.0015
	9.3	5.8	25	208.10 ± 28.79	0.572 ± 0.033	0.0055 ± 0.0005
	10.1	6.1	22	121.88 ± 3.26	0.747 ± 0.079	0.0083 ± 0.0007
	10.3	7.8	22	83.73 ± 7.15	0.634 ± 0.029	0.0096 ± 0.0007
STR	1.9	5.0	300	39.11 ± 2.80	1.472 ± 0.061	0.0013 ± 0.0001
	4.3	5.0	320	35.49 ± 1.99	3.091 ± 0.228	0.0034 ± 0.0003
	6.5	5.0	410	50.57 ± 2.40	5.047 ± 0.538	0.0051 ± 0.0006
	7.7	5.2	450	58.61 ± 3.46	5.883 ± 0.645	0.0058 ± 0.0016
	9.3	6.4	500	61.92 ± 3.78	6.460 ± 0.381	0.0088 ± 0.0017
	12.1	7.8	500	54.85 ± 0.26	7.563 ± 0.255	0.0145 ± 0.0010
